# Did Dumbo suffer a heart attack? independent association between earlobe crease and cardiovascular disease

**DOI:** 10.1186/s12872-016-0193-7

**Published:** 2016-01-20

**Authors:** Marta Aligisakis, Pedro Marques-Vidal, Idris Guessous, Peter Vollenweider

**Affiliations:** Faculty of Medicine, University of Lausanne, Lausanne, Switzerland; Department of Internal Medicine, Lausanne University Hospital (CHUV), Rue du Bugnon 46, 1011 Lausanne, Switzerland; Institute of Social and Preventive Medicine (IUMSP), Lausanne University Hospital, Lausanne, Switzerland; Department of Community Medicine, Primary Care and Emergency Medicine, Geneva University Hospitals, Geneva, Switzerland

**Keywords:** Earlobe crease, Cardiovascular diseases, Cardiovascular risk factors, Epidemiology, Switzerland

## Abstract

**Background:**

Earlobe crease (ELC) has been associated with cardiovascular diseases (CVD) or risk factors (CVRF) and could be a marker predisposing to CVD. However, most studies studied only a small number of CVRF and no complete assessment of the associations between ELC and CVRF has been performed in a single study.

**Methods:**

Population-based study (*n* = 4635, 46.7 % men) conducted between 2009 and 2012 in Lausanne, Switzerland.

**Results:**

Eight hundred six participants (17.4 %) had an ELC. Presence of ELC was associated with male gender and older age. After adjusting for age and gender (and medication whenever necessary), presence of ELC was significantly (*p* < 0.05) associated with higher levels of body mass index (BMI) [adjusted mean ± standard error: 27.0 ± 0.2 vs. 26.02 ± 0.07 kg/m^2^], triglycerides [1.40 ± 0.03 vs. 1.36 ± 0.01 mmol/L] and insulin [8.8 ± 0.2 vs. 8.3 ± 0.1 μIU/mL]; lower levels of HDL cholesterol [1.61 ± 0.02 vs. 1.64 ± 0.01 mmol/L]; higher frequency of abdominal obesity [odds ratio and (95 % confidence interval) 1.20 (1.02; 1.42)]; hypertension [1.41 (1.18; 1.67)]; diabetes [1.43 (1.15; 1.79)]; high HOMA-IR [1.19 (1.00; 1.42)]; metabolic syndrome [1.28 (1.08; 1.51)] and history of CVD [1.55 (1.21; 1.98)]. No associations were found between ELC and estimated cardiovascular risk, inflammatory or liver markers. After further adjustment on BMI, only the associations between ELC and hypertension [1.30 (1.08; 1.56)] and history of CVD [1.47 (1.14; 1.89)] remained significant. For history of CVD, further adjustment on diabetes, hypertension, total cholesterol and smoking led to similar results [1.36 (1.05; 1.77)].

**Conclusion:**

In this community-based sample ELC was significantly and independently associated with hypertension and history of CVD.

**Electronic supplementary material:**

The online version of this article (doi:10.1186/s12872-016-0193-7) contains supplementary material, which is available to authorized users.

## Background

Cardiovascular disease (CVD) is the most common cause of morbidity and mortality in industrialized countries. Most cardiovascular risk factors (CVRF) involve specific investigations (i.e. total, LDL or HDL cholesterol; glucose) and might not be suitable in low or middle-income countries, where access to health resources is scarce. Thus, the identification of simple clinical signs associated with an increased risk of CVD has drawn some attention. One of these signs is the earlobe crease (ELC), defined as “a deep crease in the lobule portion of the auricle” by S.T. Frank in 1973 [1].

Similarly to the heart, earlobes have an end-artery-type of microcirculation without collaterals and become quickly anoxic if end-arteries are occluded [2]. Therefore, CVRF associated with altered microcirculation might impact ELC due to a local micro vascular insufficiency associated with atherosclerosis [2–7].

In cross-sectional studies, ELC was associated with hypertension [8–10], obesity [2, 8], metabolic syndrome (MS) [11], atherosclerosis [8, 12], and coronary artery disease (CAD) [6, 9, 10, 13–15]. Various autopsy or biopsy studies also found an association between ELC and systemic atherosclerosis [6, 16–19] or death from CVD [20]. Further, ELC was considered an independent and early sign predisposing to CVD in several prospective studies [6, 10, 21–24]. However, some studies failed to find any association between ELC and CAD [25–27] or CVRF [13, 14, 21–23, 28] and two studies suggested that the association of CVD with ELC was entirely accounted for by increasing age [25, 29]. Some of these discrepancies might be related to the fact that most studies focused on a limited number of CVRF and none evaluated in detail the association between ELC and a large panel of established or putative CVRF such as inflammatory markers and adipokines.

Thus, the aim of this study was to assess the associations of ECL with a large panel of CVRF and also with CVD in a population-based sample of adults.

## Methods

### Recruitment

Details of the CoLaus study have been reported previously [30–32]. Briefly, CoLaus is a prospective, population-based study conducted in the city of Lausanne, Switzerland. The main aim is to better understand the prevalence of biological and genetic determinants of CVRF and CVD. The baseline assessment was conducted between 2003 and 2006 and included 6733 participants aged 35 to 75 years. The first follow-up was conducted between April 2009 and September 2012 and included 5064 participants (75.2 % of the initial sample). Non-participation was due to death in 164 (2.4 %) participants, to migration to other countries in 222 (3.3 %), to refusal in 1100 (16.3 %) and to non-traceability in 183 (2.7 %). The current results are based on the 5064 participants in the first follow-up.

The CoLaus Study was approved by the Institutional Ethics Committee of the University of Lausanne (decision 19 February 2003, protocol number 16/03). All participants provided written informed consent.

### Personal history

Personal history of CAD, angina pectoris, myocardial infarction (MI), stroke and coronary artery bypass graft (CABG) was assessed by questionnaire. Family history included MI and stroke in the mother and/or the father.

Smoking status was self-reported and defined as never, former (irrespective of the time since quitting) and current. Antihypertensive, lipid-lowering and antidiabetes medication was self-reported and defined as present or absent.

Total daily energy expenditure was assessed by a validated questionnaire (PAFQ) where the participant indicated the average daily duration of different types of physical activity (i.e. household, at work, sports) [33]. Energy expenditure was expressed in Kcalories per day and sedentary status was defined as expending less than 10 % of the daily energy in moderate- and high-intensity activities (at least 4 times the basal metabolic rate).

### Clinical data

Earlobe crease, defined as a crease in the lobule portion of the auricle, was systematically searched during the clinical examination by trained field investigators and coded as absent, unilateral or bilateral. A second coding (ELC present/absent) was also used for analysis.

Body weight and height were measured with participants standing without shoes in light indoor clothes. Body weight was measured in kilograms to the nearest 100 g using a Seca® scale (Hamburg, Germany). Height was measured to the nearest 5 mm using a Seca® gauge (Hamburg, Germany). Body mass index (BMI) was defined as weight (kg)/height (m)^2^.

Waist circumference was measured with a non-stretchable tape over the unclothed abdomen at the narrowest point between the lowest rib and the iliac crest. Two measures were made and the mean, expressed in centimetres, was used for analyses. Abdominal obesity was defined as a waist circumference >102 cm (men) and >88 cm (women) according to the National Cholesterol Education Program–Adult Treatment Panel III (NCEP-ATP III)*.*

Blood pressure was measured thrice on the left arm, with an appropriately sized cuff, after a rest of at least 10 min in the seated position using a Omron® HEM-907 automated oscillometric sphygmomanometer (OMRON, Japan). The average of the last two measurements was used for analyses. Hypertension was defined as a systolic blood pressure (SBP) >140 mmHg or a diastolic blood pressure (DBP) >90 mmHg or presence of antihypertensive medication.

### Biological data

Laboratory markers were measured in venous blood samples drawn after an overnight fast. For cytokine measurements, serum was preferred to plasma because the absolute cytokine level could be influenced by different anticoagulants [34]. Most biological assays were performed by the CHUV Clinical Laboratory on fresh blood samples within 2 h of blood collection, and additional aliquots were stored at –80 °C. Most measurements were conducted in a Modular P apparatus (Roche Diagnostics, Switzerland).

Total cholesterol was assessed by CHOD-PAP; high-density lipoprotein (HDL) cholesterol was assessed by CHOD-PAP + PEG + cyclodextrin; triglycerides were assessed by GPO-PAP; low-density lipoprotein (LDL) cholesterol was calculated using the Friedewald formula if triglycerides were <4.6 mmol/L.

Glucose was assessed by glucose dehydrogenase; insulin was assessed by a solid-phase, two-site chemiluminescent immunometric assay (Diagnostic Products Corporation, Los Angeles, USA); Homeostatic model assessment insulin resistance (HOMA-IR) was calculated as glucose (mmol/L) x insulin (μIU/mL)/ 22.5. High HOMA-IR was defined as >2.6. Diabetes was defined as a fasting plasma glucose >7.0 mmol/L or presence of antidiabetes medication.

Inflammatory markers included high sensitivity-C-reactive protein (hs-CRP), Interleukin 1β (IL-1β), interleukin 6 (IL-6) and tumour necrosis factor-α (TNF-α). Hs-CRP was assessed by immunoassay and latex HS (Immulite 1000–High, Diagnostic Products Corporation, LA, CA, USA) while the other markers were measured using a multiplexed particle-based flow cytometric cytokine assay (Millipore, Zug, Switzerland). Lower detection limits for IL-1β, IL-6 and TNF-α were 0.2 ng/L. As many participants had cytokine levels below detection limits (28 % for IL-1β and 4.5 % for IL-6), inflammatory markers were categorized into quartiles and undetectable values were included in the first quartile. Log-transformed values of available levels were also used in the analysis.

Leptin was measured by ELISA (American Laboratory Products Company, Windham, USA). Adiponectin was assessed by ELISA (R&D Systems, Inc, Minneapolis, USA). Uric acid was assessed by uricase-PAP and creatinine was assessed by the Jaffe kinetic compensated method.

Aspartate aminotransferase (ASAT), alanine aminotransferase (ALAT), γ-glutamyl transpeptidase (γ-GT) and alkaline phosphatase were assessed by the optimized standard method according to the International Federation of Clinical Chemistry at 37 °C.

### Other variables

Metabolic syndrome was defined according to the NCEP ATP-III as at least three of the following criteria: 1) waist circumference >102 cm (men) and >88 cm (women), 2) triglycerides ≥1.7 mmol/L and/or HDL cholesterol <1.03 mmol/L (men) or <1.29 mmol/L (women) or lipid lowering medication, 3) Blood pressure ≥130/85 mm Hg or antihypertensive medication, 4) Fasting plasma glucose ≥5.6 mmol/L or antidiabetes medication.

Cardiovascular risk was estimated using three different equations: a calibrated SCORE [35], the original Framingham 1998 equation, and a calibrated version of the Framingham 1998 equation for the Swiss population [36].

### Statistical analysis

Statistical analyses were performed using Stata version 13.1 for Windows (Stata Corp, College Station, Texas, USA). Bivariate statistical analyses were performed by Student’s t-test for quantitative variables and by chi-square for categorical variables. Results were expressed as mean ± standard deviation or as number of participants (percentage). Sensitivity, specificity, positive and negative predictive values for presence versus absence of ELC were computed and presented with their 95 % confidence intervals. For quantitative variables, multivariable analyses were performed by ANOVA and the results were expressed as adjusted mean ± standard error. For categorical variables (excluding smoking), multivariable analyses were performed by logistic regression and results were expressed as odds ratio (OR) and 95 % confidence interval. For smoking, multivariable analyses were performed using multivariate (polytomous) logistic regression and the results were expressed as relative risk ratio (95 % confidence interval). Two models were applied: adjusted for 1) age and gender and 2) age, gender, and BMI. Analyses were also adjusted for medication whenever appropriate. As cardiovascular risk equations already include cardiovascular risk factors, no adjustment was performed for CVRF. For sensitivity analysis, a model similar to 2) but adjusting for waist circumference instead of BMI was applied; similarly, for history of CVD a further adjustment for hypertension, diabetes, total cholesterol and smoking was performed. Statistical significance was assessed for a two-sided test *p*-value <0.05.

## Results

### Characteristics of participants

Of the 5064 participants of the first follow-up, 429 (8.5 %) were excluded because of missing data regarding ELC. Excluded participants were older, had a higher SBP and presented more often with hypertension and abdominal obesity than included participants; the other main characteristics and biological markers did not differ between the two groups (Additional file 1: Table S1). The remaining 4635 participants (46.7 % men) had a mean age of 57.8 ± 10.5 years and a mean BMI of 26.2 ± 4.6 kg/m^2^. Eight hundred and six (17.4 %) participants had an ELC, of which 373 (46.3 %) unilateral.

### Association between ELC and CVRF

On bivariate analysis, presence of ELC was significantly associated with higher BMI, SBP, DBP, triglyceride, glucose and insulin levels, and with lower HDL cholesterol levels. Presence of ELC was also associated with a higher likelihood of presenting with abdominal obesity, hypertension, diabetes, insulin resistance or metabolic syndrome. Conversely, no association was found between ELC and smoking status, total and LDL cholesterol (Table 1). Sensitivity, specificity, positive and negative predictive values for presence versus absence of ELC regarding cardiovascular risk factors are presented in Additional file 2: Table S2. For all risk factors sensitivity and positive predictive values were low, whereas specificity and negative predictive values were relatively high, namely regarding personal history of CVD (Additional file 2: Table S2).

**Table 1:** Bivariate analysis of the association between earlobe crease and selected cardiovascular risk factors (CoLaus study, Lausanne, 2009-2012)

Earlobe crease	Absence (*n* = 3829)	Presence (*n* = 806)	*P*-value
Men (%)	1708 (44.6)	455 (56.5)	<0.001
Age (years)	56.3 ± 10.3	63.2 ± 9.7	<0.001
BMI (kg/m2)	25.94 ± 4.53	27.38 ± 4.85	<0.001
BMI categories (%)			
Normal	1749 (46.2)	255 (32.2)	
Overweight	1448 (38.2)	349 (44.1)	<0.001
Obese	592 (15.6)	187 (23.6)	
Abdominal obesity (%)	1382 (36.3)	362 (45.2)	<0.001
Smoking status (%)			
Never	1619 (42.3)	300 (37.2)	
Former	1358 (35.5)	347 (45.1)	<0.001
Current	852 (22.2)	159 (19.7)	
Blood pressure status			
SBP (mm Hg)	125 ± 17	131 ± 19	<0.001
DBP (mm Hg)	78 ± 11	79 ± 11	<0.001
Hypertension (%)	1409 (36.9)	475 (58.9)	<0.001
Lipids (mmol/L)			
Total cholesterol	5.70 ± 1.03	5.68 ± 1.10	0.66
LDL cholesterol	3.45 ± 0.92	3.44 ± 0.98	0.88
HDL cholesterol	1.65 ± 0.47	1.59 ± 0.47	<0.001
Triglycerides	1.34 ± 0.90	1.47 ± 0.91	<0.001*
Glycaemic status			
Glucose (mmol/L)	5.83 ± 1.06	6.20 ± 1.59	<0.001
Insulin (μIU/mL)	8.06 ± 6.82	9.75 ± 7.74	<0.001*
Diabetes (%)	343 (9.0)	147 (18.3)	<0.001
HOMA-IR	2.21 ± 2.48	2.86 ± 2.65	<0.001
High HOMA-IR (%)	925 (24.2)	277 (34.4)	<0.001
Metabolic syndrome (%)	1070 (28.2)	337 (42.1)	<0.001

Results are expressed as number of participants (%) or as mean ± standard deviation. Statistical analysis by Student’s t-test or by chi-square

*BMI* body mass index; abdominal obesity and metabolic syndrome are defined by the NCEP ATP-III criteria; hypertension is defined as SBP > 140 or DBP > 90 mm Hg and/or anti-hypertensive medication; *LDL* low-density lipoprotein; *HDL* high-density lipoprotein; *HOMA-IR* homeostatic model assessment of insulin resistance; high HOMA-IR is defined as ≥2.6; diabetes is defined as fasting plasma glucose >7.0 mmol/L and/or anti-diabetes medication

*P-value calculated on log-transformed values

After multivariable adjustment on age, gender and medication (whenever necessary), the associations of ELC with BMI, abdominal obesity, hypertension, glucose and insulin levels, diabetes, HOMA-IR, and MS remained (Table 2). Similarly, significant associations were found between the number of ELC and BMI, hypertension, HDL cholesterol, insulin levels, HOMA-IR and diabetes (Additional file 3: Table S3 and Additional file 4: Table S4). After further adjustment for BMI, only hypertension remained associated with ELC, while no association was found with the number of ELC. Similar findings were obtained when the analysis was adjusted for waist instead of BMI (Additional file 5: Table S5).

**Table 2:** Multivariable analysis of the association between earlobe crease and selected cardiovascular risk factors, CoLaus study, Lausanne, 2009–2012

Adjusted for	Age and gender			Age, gender and body mass index		
Earlobe crease	Absence (*n* = 3829)	Presence (*n* = 806)	*P*-value	Absence (*n* = 3829)	Presence (*n* = 806)	*P*-value
BMI (kg/m^2^)	26.0 ± 0.1	27.0 ± 0.2	<0.001	–	–	–
Abdominal obesity	1 (ref.)	1.20 (1.02; 1.42)	<0.05	1 (ref.)	0.85 (0.66; 1.09)	0.19
Smoking status						
Never	–	–	–	–	–	–
Former	1 (ref.)	1.17 (0.98; 1.40)	0.08	1 (ref.)	1.15 (0.96; 1.37)	0.14
Current	1 (ref.)	1.11 (0.89; 1.38)	0.34	1 (ref.)	1.16 (0.93; 1.45)	0.18
Blood pressure status						
SBP (mm Hg)^a^	126 ± 1	126 ± 1	0.43	126 ± 1	126 ± 1	0.89
DBP (mm Hg)^a^	78 ± 1	79 ± 1	0.11	78 ± 1	78 ± 1	0.53
Hypertension	1 (ref.)	1.41 (1.18; 1.67)	<0.001	1 (ref.)	1.30 (1.08; 1.56)	<0.01
Lipids (mmol/L)^b^						
Total cholesterol	5.70 ± 0.02	5.68 ± 0.04	0.69	5.70 ± 0.02	5.68 ± 0.04	0.53
LDL cholesterol	3.45 ± 0.01	3.45 ± 0.03	0.94	3.45 ± 0.01	3.43 ± 0.03	0.57
HDL cholesterol	1.64 ± 0.01	1.61 ± 0.02	0.05	1.64 ± 0.01	1.64 ± 0.01	0.93
Triglycerides	1.36 ± 0.01	1.40 ± 0.03	0.05*	1.36 ± 0.01	1.36 ± 0.03	0.65*
Glycaemic status						
Glucose (mmol/L)^c^	5.88 ± 0.02	5.96 ± 0.04	0.06	5.88 ± 0.02	5.92 ± 0.04	0.36
Insulin (μIU/mL)^c^	8.3 ± 0.1	8.8 ± 0.2	0.01*	8.3 ± 0.1	8.4 ± 0.2	0.78*
HOMA-IR^c^	2.29 ± 0.04	2.47 ± 0.09	0.07	2.30 ± 0.04	2.33 ± 0.08	0.72
Diabetes	1 (ref.)	1.43 (1.15; 1.79)	<0.01	1 (ref.)	1.25 (0.99; 1.59)	0.07
High HOMA-IR	1 (ref.)	1.19 (1.00; 1.42)	<0.05	1 (ref.)	0.95 (0.78; 1.16)	0.62
Metabolic syndrome	1 (ref.)	1.28 (1.08; 1.51)	<0.01	1 (ref.)	1.03 (0.84; 1.26)	0.79

For quantitative variables, multivariable analyses were performed by ANOVA and the results are expressed as adjusted mean ± standard error. For categorical variables (excluding smoking), multivariable analyses were performed by logistic regression and results are expressed as odds ratio (95 % confidence interval). For smoking, multivariable analyses were performed using multivariate (polytomous) logistic regression and the results are expressed as relative risk ratio (95 % confidence interval)

*BMI* body mass index; *SBP* systolic blood pressure; *DBP* diastolic blood pressure; *LDL* low-density lipoprotein; *HDL* high-density lipoprotein; *HOMA-IR* homeostatic model assessment of insulin resistance; high HOMA-IR is defined as a HOMA-IR ≥ 2.6; abdominal obesity and metabolic syndrome are defined by the NCEP ATP-III criteria; hypertension is defined as SBP > 140 or DBP > 90 mm Hg or anti-hypertensive medication; diabetes is defined as fasting plasma glucose >7.0 mmol/L or antidiabetes medication

*P-value calculated on log-transformed values

^a^,adjusting for antihypertensive medication

^b^,adjusting for hypolipidemic medication

^c^,adjusting for antidiabetes medication

### Association between ELC and history or risk of CVD

The prevalence of ELC was 16 % in participants without any history of CVD and 33.3 % in those with anamnestic CVD (*p* value < 0.001).

On bivariate analysis, the presence of an ELC was associated with personal history of CVD (CAD; MI; angina pectoris; stroke) or CABG, and with a higher CVD risk (Table 3). Most associations with personal history of CVD remained significant after multivariable adjustment, while the associations with CVD risk became non-significant (Tables 4). Similar findings were obtained when the analysis was adjusted for waist instead of BMI (Additional file 6: Table S6) or when the analysis was adjusted for other CVRF (Additional file 7: Table S7). Conversely, no association was found between ELC and family history of CVD (not shown).

**Table 3:** Bivariate analysis of the association between earlobe crease and history or risk of cardiovascular disease, CoLaus study, Lausanne, 2009–2012

Earlobe crease	Absence (*n* = 3829)	Presence (*n* = 806)	*P*-value
History of			
Any cardiovascular disease	244 (6.4)	122 (15.1)	<0.001
Coronary artery disease	92 (2.4)	60 (7.5)	<0.001
Angina pectoris	61 (1.6)	30 (3.7)	<0.001
Myocardial infarction	60 (1.6)	34 (4.2)	<0.001
Stroke	61 (1.6)	28 (3.5)	<0.001
Coronary artery bypass graft	33 (0.9)	22 (2.7)	<0.001
High cardiovascular disease risk			
SCORE recalibrated	466 (12.2)	221 (27.5)	<0.001
Framingham 1998	355 (9.3)	161 (20.0)	<0.001
Framingham 1998 recalibrated	453 (11.8)	201 (24.9)	<0.001

Results are expressed as number of participants (column %). Statistical analysis by chi-square. High cardiovascular disease risk is defined as a 10-year risk ≥5 % for SCORE and as a 10-year risk ≥20 % for Framingham

**Table 4:** Multivariable analysis of the association between earlobe crease and personal history of cardiovascular disease, CoLaus study, Lausanne, 2009–2012

Adjusted for	Age and gender		Age, gender and BMI	
History of	OR (95 % CI)	*P*-value	OR (95 % CI)	*P*-value
Any CVD	1.55 (1.21; 1.98)	<0.001	1.47 (1.14; 1.89)	<0.01
Coronary artery disease	1.85 (1.30; 2.63)	<0.001	1.80 (1.26; 2.58)	<0.001
Angina pectoris	1.28 (0.81; 2.03)	0.30	1.19 (0.74; 1.90)	0.47
Myocardial infarction	1.69 (1.08; 2.64)	<0.05	1.59 (1.01; 2.51)	<0.05
Stroke	1.34 (0.84; 2.15)	0.23	1.30 (0.79; 2.11)	0.30
CABG	1.70 (0.97; 3.00)	0.07	1.67 (0.93; 2.99)	0.09
High CVD risk				
SCORE recalibrated	1.11 (0.88; 1.41)	0.37	1.10 (0.87; 1.39)	0.44
Framingham 1998	1.09 (0.85; 1.41)	0.50	0.99 (0.76; 1.28)	0.91
Framingham 1998 r.	1.15 (0.91; 1.47)	0.25	1.04 (0.81; 1.34)	0.75

Results are expressed as adjusted odds ratio (95 % confidence interval) for presence relative to the absence of ELC. Statistical analysis by logistic regression

*BMI* body mass index; *OR* odds ratio; *CVD* cardiovascular disease; *CABG* coronary artery bypass graft; high cardiovascular disease risk defined as a 10-year risk ≥5 % for SCORE and as a 10-year risk ≥20 % for Framingham. r. recalibrated

Similar associations between ELC and personal history of CVD were obtained after splitting the participants with ELC on unilateral and bilateral ELC (Additional file 6: Table S6, Additional file 7: Table S7, Additional file 8: Table S8, Additional file 9: Table S9).

### Association between ELC and other markers

On bivariate analysis, presence of ELC was associated with higher levels of leptin, uric acid, creatinine and liver enzymes, while no association was found with energy expenditure, sedentary status and adiponectin levels (Additional file 10: Table S10). All associations became non-significant after multivariable adjustment (Additional file 11: Table S11) and similar findings were obtained when adjusting for waist instead of BMI (Additional file 12: Table S12).

On bivariate analysis, presence of ELC was positively associated with Hs-CRP and TNF-α, but not with IL-1β or IL-6 (Additional file 13: Table S13). After multivariable adjustment, a positive association was found between ELC and IL-1β in quartiles, while no association was found for the other inflammatory markers (Additional file 14: Table S14). Similar results were obtained when the analysis was performed on log-transformed values, except that the association between ELC and Il-1β was no longer significant (Additional file 14: Table S14) or when the analysis was adjusted for waist instead of BMI (Additional file 15: Table S15).

## Discussion

To our knowledge, this is the most comprehensive study on the associations between ELC and CVRF. Our results show that ELC is significantly associated with hypertension and history of CVD independently of other risk factors or potential confounders. Our results also suggest that the associations with the other CVRF reported in the literature could be due to confounding by age, gender or BMI.

### Association between ELC and CVRF

Prevalence of ELC was higher in men and increased with age, a finding already reported [10, 11, 13, 26, 37]. A possible explanation is the changes in skin collagen due to aging, which would lead to the development of ELC [2–7]. The higher prevalence of ELC in men could be related to a higher prevalence of smoking, which would accelerate the skin aging process. Still, no associations between ELC and smoking status were found, a finding in agreement with several other studies [2, 7, 8, 21], but not with another [10]. Thus, the higher prevalence of ELC in men remains an open question.

ELC was strongly and linearly associated with higher BMI and abdominal obesity, a finding already reported by two studies [2, 8] but not by another [10]. Interestingly, most associations between ELC and CVRF or CVD were no longer significant after adjusting for BMI, suggesting that ELC might be a stronger marker of obesity than of other CVRF. The fact that the associations between ELC and hypertension, glucose level and history of CAD remained significant after adjusting for BMI also suggests that ELC could be used as a marker of these CVRF.

ELC was positively associated with hypertension, and this association remained significant even after adjusting for age, gender and BMI. Such association had already been pointed out by some reports [8–10] but by another [13]. Contrary to BMI, no linear association between number of ELC and prevalence of hypertension was found.

On bivariate analysis, ELC was associated with diabetes and several markers of insulin resistance such as lower HDL cholesterol and higher glucose, insulin, HOMA-IR and triglycerides levels. Still, all associations became non-significant after adjusting for BMI, a finding partly in agreement with other studies [7, 8, 13]. Similarly, the association between ELC and MS was no longer significant after adjusting for BMI, a finding contradicting another study which found a high association between ELC and MS [11]. Overall, our results suggest that the association between ELC and most metabolic markers might be mediated by obesity levels.

### Association between ELC and history or risk of CVD

ELC was significantly associated with personal history of CVD (particularly CAD) independently of age, gender and BMI; adjusting for waist circumference instead of BMI or further adjustment for the main CVRF (hypertension, smoking, total cholesterol and diabetes) led to similar findings. Interestingly, the fact of having bilateral ELC did not lead to a stronger association than having unilateral ELC. Conversely, no association was found between ELC and CVD risk scores after adjusting for age, gender and BMI; the most likely explanation is that age and gender are part of CVD risk equations and that participants with ELC were older, thus leading to an increased CVD risk. Overall, our results suggest that ELC might be related to personal history of CVD but not to absolute CVD risk.

The main hypothesis to explain the association between ELC and CVD is that both organs have an end-artery-type of microcirculation without collaterals [2–7]. Thus, atherosclerotic microvascular changes leading to anoxia in earlobes and to the occurrence of ELC could reflect changes in heart microcirculation. In other words, presence of an ELC would be a marker of defective heart microcirculation. Our findings of an independent association with hypertension which also has a negative impact on the microcirculation are consistent with this hypothesis.

### Association between ELC and other markers

Previous studies have explored the association between ELC and obesity or MS [2, 8, 11] but data on the associations between ELC and other adiposity markers such as leptin, adiponectin and uric acid are lacking. Our results show that most bivariate associations between ELC and metabolic, adiposity, inflammatory or liver markers were no longer significant in multivariable analysis.

Still, a significant positive association was found between ELC and IL-1β, independently of age, gender and BMI or waist. IL-1β is a pro-inflammatory cytokine and has been suggested to be involved in the pathogenesis of atherosclerosis and vascular and myocardial injury [38–40]. However, in the CoLaus study IL-1β levels were not associated with obesity [41].

### Study limitations

This study has several limitations. Due to its cross-sectional setting, it was not possible to estimate whether presence of ELC is an independent predictor of CVD. A follow-up study of this cohort is currently under way and will hopefully provide an answer to this issue. Finally, this study is based on a Caucasian population and the results might not be applied to other ethnic groups.

## Conclusion

Earlobe crease is significantly and independently associated with higher prevalence of hypertension and personal history of CVD. The association between ELC and the other metabolic markers appears to be mediated by obesity.

## Availability of supporting data

Researchers using the data are required to follow the terms of a Data Transfer Agreement containing a number of clauses designed to ensure protection of privacy and compliance with relevant laws. For further information go to http://www.colaus.ch/en/cls_home/cls_pro_home/cls-research-3/cls-agreement.htm. All data requests should be sent to the principal investigator of the CoLaus study.

## Additional files

Additional file 1: Table S1. Comparison between included and excluded participants, CoLaus study, Lausanne, 2009-2012. (PDF 400 kb) Additional file 2: Table S2. Sensitivity analysis of presence of earlobe crease regarding cardiovascular risk factors and cardiovascular disease, CoLaus study, Lausanne, 2009-2012. (PDF 41 kb) Additional file 3: Table S3. Bivariate analysis of the association between earlobe crease unilateral or bilateral and selected cardiovascular risk factors, CoLaus study, Lausanne, 2009–2012. (PDF 49 kb) Additional file 4: Table S4. Multivariable analysis of the association between earlobe crease (unilateral or bilateral) and selected cardiovascular risk factors, CoLaus study, Lausanne, 2009–2012. (PDF 63 kb) Additional file 5: Table S5. Multivariable analysis of the association between earlobe crease and selected cardiovascular risk factors, CoLaus study, Lausanne, 2009–2012, adjusting for age, gender and waist circumference. (PDF 59 kb) Additional file 6: Table S6. Multivariable analysis of the association between earlobe crease and personal history of cardiovascular disease or cardiovascular risk, CoLaus study, Lausanne, 2009–2012, adjusting for age, gender and waist circumference. (PDF 51 kb) Additional file 7: Table S7. Multivariable analysis of the association between earlobe crease and personal history of cardiovascular disease, CoLaus study, Lausanne, 2009–2012, adjusted for age, gender, waist circumference, total cholesterol, hypertension, diabetes and smoking. (PDF 46 kb) Additional file 8: Table S8. Bivariate association between earlobe crease (unilateral or bilateral) and history of cardiovascular disease, CoLaus study, Lausanne, 2009–2012. (PDF 39 kb) Additional file 9: Table S9. Multivariable association between earlobe crease (unilateral or bilateral) and history of cardiovascular disease, CoLaus study, Lausanne, 2009–2012. (PDF 48 kb) Additional file 10: Table S10. Bivariate analysis of the association between earlobe crease and physical activity, adipokines or liver markers, CoLaus study, Lausanne, 2009–2012. (PDF 43 kb) Additional file 11: Table S11. Multivariable analysis of the association between ELC and physical activity, adipokines or liver markers, CoLaus study, Lausanne, 2009–2012. (PDF 54 kb) Additional file 12: Table S12. Multivariable analysis of the association between earlobe crease and physical activity, adipokines or liver markers, CoLaus study, Lausanne, 2009–2012, adjusted for age, gender and waist circumference. (PDF 55 kb) Additional file 13: Table S13. Bivariate association between earlobe crease and quartiles of inflammatory markers, CoLaus study, Lausanne, 2009–2012. (PDF 41 kb) Additional file 14: Table S14. Multivariable association between earlobe crease and being in the highest quartile of inflammatory markers, CoLaus study, Lausanne, 2009–2012. (PDF 51 kb) Additional file 15: Table S15. Multivariable association between earlobe crease and being in the highest quartile of inflammatory markers, CoLaus study, Lausanne, 2009–2012, adjusted for age, gender and waist circumference. (PDF 57 kb)

## Abbreviations

ALAT: Alanine aminotransferase
ASAT: Aspartate aminotransferase
BMI: Body mass index
CABG: Coronary artery bypass graft
CAD: Coronary artery disease
CVD: Cardiovascular diseases
CVRF: Risk factors
DBP: Diastolic blood pressure
ELC: Earlobe crease
HDL: High-density lipoprotein cholesterol
HOMA-IR: Homeostatic model assessment insulin resistance
hs-CRP: High sensitivity-C-reactive protein
IL-1β: Interleukin 1β
IL-6: Interleukin 6
LDL: Low-density lipoprotein cholesterol
MI: Myocardial infarction
MS: Metabolic syndrome
NCEP-ATP III: National Cholesterol Education Program–Adult Treatment Panel III
OR: Odds ratio
SBP: Systolic blood pressure
TNF-α: Tumour necrosis factor-α
γ-GT: γ-glutamyl transpeptidase
