# Supplementary material for: Did Dumbo suffer a heart attack? independent association between earlobe crease and cardiovascular disease
Source: BMC Cardiovasc Disord. 2016 Jan 20;16:17. doi: 10.1186/s12872-016-0193-7 (PMC4721195; doi:10.1186/s12872-016-0193-7)
Supplement: Additional file 1: Table S1. — Comparison between included and excluded participants, CoLaus study, Lausanne, 2009-2012. (PDF 400 kb) [file 12872_2016_193_MOESM1_ESM.pdf]

Supplementary table 1: Comparison between included and excluded participants, CoLaus study, Lausanne, 2009-2012.

	Included (N=4635)	Excluded (N=429)	P-value
Men (%)	2163 (46.7)	194 (45.2)	0.57
Age (years)	57.5 ± 10.5	61.0 ± 10.2	<0.001
BMI (kg/m^2)	26.2 ± 4.6	26.3 ± 4.6	0.64
BMI categories (%)			
Normal	2004 (43.8)	176 (42.0)	
Overweight	1797 (39.2)	163 (38.9)	0.54
Obese	779 (17.0)	80 (19.1)	
Abdominal obesity (%)	1744 (37.8)	194 (45.9)	<0.001
Smoking status (%)			
Never	1919 (41.4)	173 (40.3)	
Former	1705 (36.8)	178 (41.5)	0.09
Current	1011 (21.8)	78 (18.2)	
Blood pressure status			
SBP (mm Hg)	126 ± 18	129 ± 19	0.003
DBP (mm Hg)	78 ± 11	78 ± 11	0.54
Hypertension (%)	1884 (40.7)	212 (49.9)	<0.001
Lipids (mmol/L)			
Total cholesterol	5.7 ± 1.0	5.7 ± 1.1	0.61
LDL cholesterol	3.4 ± 0.9	3.4 ± 0.9	0.74
HDL cholesterol	1.6 ± 0.5	1.6 ± 0.5	0.84
Triglycerides	1.4 ± 0.9	1.3 ± 0.8	0.80 §
Glycaemic status			
Glucose (mmol/L)	5.9 ± 1.2	5.8 ± 1.1	0.41
Insulin (µIU/mL)	8.4 ± 7.1	8.8 ± 7.2	0.16 §
Diabetes (%)	490 (10.6)	49 (11.6)	0.53
HOMA-IR	2.3 ± 2.6	2.4 ± 2.4	0.59
High HOMA-IR (%)	1202 (25.9)	115 (26.8)	0.69
Metabolic syndrome (%)	1407 (30.7)	141 (33.7)	0.19

Results are expressed as number of participants (%) or as mean ± standard deviation. Statistical analysis by Student's t-test or by chi-square. § P-value calculated on log-transformed values.

BMI, body mass index; **abdominal obesity** and **metabolic syndrome** are defined by the NCEP ATP-III criteria; **hypertension** is defined as SBP>140 or DBP>90 mm Hg or antihypertensive medication; **LDL**, low-density lipoprotein; **HDL**, high-density lipoprotein; **HOMA-IR**, homeostatic model assessment of insulin resistance; **high HOMA-IR** is defined as a HOMA-IR≥2.6; **diabetes** is defined as fasting plasma glucose >7.0 mmol/L or antidiabetes medication.
