# Supplementary material for: Did Dumbo suffer a heart attack? independent association between earlobe crease and cardiovascular disease
Source: BMC Cardiovasc Disord. 2016 Jan 20;16:17. doi: 10.1186/s12872-016-0193-7 (PMC4721195; doi:10.1186/s12872-016-0193-7)
Supplement: Additional file 2: Table S2. — Sensitivity analysis of presence of earlobe crease regarding cardiovascular risk factors and cardiovascular disease, CoLaus study, Lausanne, 2009-2012. (PDF 41 kb) [file 12872_2016_193_MOESM2_ESM.pdf]

Supplementary table 2 : Sensitivity analysis of presence of earlobe crease regarding cardiovascular risk factors and cardiovascular disease, CoLaus study, Lausanne, 2009-2012.

	**Sensitivity**	**Specificity**	**PPV**	**NPV**
Risk factors				
Abdominal obesity	20.8 (18.9 - 22.7)	84.7 (83.3 – 86.0)	45.2 (41.7 - 48.7)	63.7 (62.2 - 65.3)
Hypertension	25.2 (23.3 - 27.2)	87.9 (86.7 - 89.1)	58.9 (55.4 - 62.4)	63.1 (61.6 - 64.7)
Diabetes	30.0 (26.0 - 34.3)	84.1 (82.9 - 85.2)	18.3 (15.7 - 21.1)	91.0 (90.1 - 91.9)
High HOMA-IR	23.0 (20.7 - 25.5)	84.6 (83.3 - 85.8)	34.4 (31.1 - 37.8)	75.8 (74.5 - 77.2)
Metabolic syndrome	24.0 (21.7 - 26.3)	85.5 (84.2 - 86.7)	42.1 (38.7 - 45.6)	71.8 (70.3 - 73.2)
History of				
Any CVD	33.3 (28.5 - 38.4)	84.0 (82.8 - 85.1)	15.1 (12.7 - 17.8)	93.6 (92.8 - 94.4)
CAD	39.5 (31.6 - 47.7)	83.4 (82.3 - 84.5)	7.5 (5.8 - 9.5)	97.6 (97.1 - 98.1)
Angina pectoris	33.0 (23.5 - 43.6)	82.9 (81.8 - 84.0)	3.7 (2.5 - 5.3)	98.4 (97.9 - 98.8)
Myocardial infarction	36.2 (26.5 - 46.7)	83.0 (81.9 - 84.1)	4.2 (3.0 - 5.9)	98.4 (98.0 - 98.8)
Stroke	31.5 (22.0 - 42.2)	82.9 (81.8 – 84.0)	3.5 (2.3 – 5.0)	98.4 (98.0 - 98.8)
CABG	40.0 (27.0 - 54.1)	82.9 (81.7 - 83.9)	2.7 (1.7 - 4.1)	99.1 (98.8 - 99.4)
High CVD risk				
SCOREr.	32.2 (28.7 - 35.8)	85.1 (84.0 - 86.2)	27.5 (24.4 - 30.7)	87.8 (86.7 - 88.8)
Framingham 1998	31.2 (27.2 - 35.4)	84.3 (83.2 - 85.4)	20.0 (17.3 - 22.9)	90.7 (89.8 - 91.6)
Framingham 1998r.	30.7 (27.2 - 34.4)	84.8 (83.6 - 85.9)	24.9 (22.0 - 28.1)	88.2 (87.1 - 89.2)

Results are expressed as value and (95% confidence interval). The area under the curve (receiver-operating curve or ROC) can be computed as (Sensitivity + Specificity)/2.

PPV, positive predictive value ; **NPV**, negative predictive value ; **HOMA-IR**, homeostatic model assessment of insulin resistance. **High HOMA-IR** is defined as ≥2.6; **abdominal obesity** and **metabolic syndrome** are defined by the NCEP ATP-III criteria; **hypertension** is defined as SBP>140 or DBP>90 or anti-hypertensive medication; **diabetes** is defined as fasting plasma glucose >7.0mmol/L or anti-diabetes medication; **CVD**, cardiovascular diseases; **CAD**, coronary artery disease; **CABG**, Coronary artery bypass graft; **high CVD risk** defined as ≥5%. r.; recalibrated.
