# Supplementary material for: Did Dumbo suffer a heart attack? independent association between earlobe crease and cardiovascular disease
Source: BMC Cardiovasc Disord. 2016 Jan 20;16:17. doi: 10.1186/s12872-016-0193-7 (PMC4721195; doi:10.1186/s12872-016-0193-7)
Supplement: Additional file 3: Table S3. — Bivariate analysis of the association between earlobe crease unilateral or bilateral and selected cardiovascular risk factors, CoLaus study, Lausanne, 2009–2012. (PDF 49 kb) [file 12872_2016_193_MOESM3_ESM.pdf]

Supplementary table 3: Bivariate analysis of the association between earlobe crease unilateral or bilateral and selected cardiovascular risk factors, CoLaus study, Lausanne, 2009-2012.

Earlobe crease	Absent (n=3829)	Unilateral (n=373)	Bilateral (n=429)	P-value
Men (%)	1708 (44.6)	234 (62.7)	217 (50.6)	<0.001
Age (years)	56.3 ± 10.3	62.0 ± 9.5	64.2 ± 9.8	<0.001
BMI (kg/m^2)	25.94 ± 4.53	27.17 ± 4.75	27.56 ± 4.94	<0.001
BMI categories (%)				
Normal	1749 (87.3)	130 (6.5)	125 (6.2)	
Overweight	1448 (80.6)	150 (8.4)	199 (11.1)	<0.001
Obese	592 (76.0)	89 (11.4)	98 (12.6)	
Abdominal obesity (%)	1382 (36.3)	157 (42.3)	205 (47.7)	<0.001
Smoking status (%)				
Never	1358 (79.7)	150 (8.8)	197 (11.6)	
Former	1581 (84.2)	153 (8.2)	144 (7.7)	<0.001
Current	852 (84.3)	70 (6.9)	89 (8.8)	
Blood pressure status				
SBP (mm Hg)	125 ± 17	131 ± 17	132 ± 20	<0.001
DBP (mm Hg)	78 ± 11	80 ± 11	79 ± 12	<0.001
Hypertension (%)	1409 (36.9)	215 (57.6)	260 (60.1)	<0.001
Lipids (mmol/L)				
Total cholesterol	5.70 ± 1.03	5.67 ± 1.04	5.70 ± 1.15	0.83
LDL cholesterol	3.45 ± 0.92	3.43± 0.92	3.45± 1.03	0.94
HDL cholesterol	1.65 ± 0.47	1.58 ± 0.45	1.59 ± 0.48	<0.01
Triglycerides	1.34 ± 0.90	1.48 ± 0.96	1.46 ± 0.85	<0.001 §
Glycaemic status				
Glucose (mmol/L)	5.83 ± 1.06	6.16 ± 1.56	6.22 ± 1.62	<0.001
Insulin (µIU/mL)	8.06 ± 6.86	9.67 ± 8.22	9.82 ± 7.32	<0.001 §
Diabetes (%)	343 (9.0)	67 (18.0)	80 (18.6)	<0.001
HOMA-IR	2.21 ± 2.48	2.74 ± 2.67	2.95 ± 3.13	<0.001
High HOMA-IR (%)	925 (24.2)	129 (34.6)	148 (34.2)	<0.001
Metabolic syndrome (%)	1070 (28.2)	156 (42.1)	181 (42.2)	<0.001

Results are expressed as number of participants (%) or as mean ± standard deviation. Statistical analysis by Student's t-test or by chi-square. § P-value calculated on log-transformed values.

BMI, body mass index; **abdominal obesity** and **metabolic syndrome** are defined by the NCEP ATP-III criteria; **hypertension** is defined as SBP>140 or DBP>90 mm Hg or antihypertensive medication; **LDL**, low-density lipoprotein; **HDL**, high-density lipoprotein; **HOMA-IR**, homeostatic model assessment of insulin resistance; **high HOMA-IR** is defined as a HOMA-IR ≥2.6; **diabetes** is defined as fasting plasma glucose >7.0 mmol/L or antidiabetes medication.
