# Supplementary material for: Did Dumbo suffer a heart attack? independent association between earlobe crease and cardiovascular disease
Source: BMC Cardiovasc Disord. 2016 Jan 20;16:17. doi: 10.1186/s12872-016-0193-7 (PMC4721195; doi:10.1186/s12872-016-0193-7)
Supplement: Additional file 4: Table S4. — Multivariable analysis of the association between earlobe crease (unilateral or bilateral) and selected cardiovascular risk factors, CoLaus study, Lausanne, 2009–2012. (PDF 63 kb) [file 12872_2016_193_MOESM4_ESM.pdf]

Supplementary table 4: Multivariable analysis of the association between earlobe crease (unilateral or bilateral) and selected cardiovascular risk factors, CoLaus study, Lausanne, 2009-2012.

**Adjusting for**	**Age and gender**				**Age, gender and body mass index**			
**Earlobe crease**	**Absent (n=3829)**	**Unilateral (n=373)**	**Bilateral (n=429)**	**P-value for trend**	**Absent (n=3829)**	**Unilateral (n=373)**	**Bilateral (n=429)**	**P-value for trend**
BMI (kg/m²)	26.0 ± 0.1	26.8 ± 0.2	27.2 ± 0.2	<0.001	-	-	-	-
Abdominal obesity	1 (ref.)	1.17 (0.93; 1.46)	1.24 (1.00; 1.53)	0.05	1 (ref.)	0.95 (0.67; 1.33)	0.78 (0.57; 1.06)	0.12
Smoking status								
Never	-							
Former	1 (ref.)	0.97 (0.76; 1.24)	1.38 (1.10; 1.75)	NA	1 (ref.)	0.97 (0.76; 1.24)	1.33 (1.05; 1.69)	NA
Current	1 (ref.)	0.91 (0.67; 1.23)	1.33 (1.00; 1.77)	NA	1 (ref.)	0.96 (0.71; 1.30)	1.39 (1.04; 1.86)	NA
Blood pressure status								
SBP (mm Hg) [a]	126 ± 1	126 ± 1	127 ± 1	0.18	126 ± 1	125 ± 1	127 ± 1	0.37
DBP (mm Hg) [a]	78 ± 1	79 ± 1	78 ± 1	0.43	78 ± 1	79 ± 1	78 ± 1	0.89
Hypertension	1 (ref.)	1.41 (1.11; 1.79)	1.40 (1.12; 1.76)	0.003	1 (ref.)	1.34 (1.05; 1.72)	1.26 (1.00; 1.6)	0.054
Lipids (mmol/L) [b]								
Total cholesterol	5.70 ± 0.02	5.69 ± 0.05	5.68 ± 0.05	0.64	5.70 ± 0.02	5.69 ± 0.05	5.66 ± 0.05	0.47
LDL cholesterol	3.45 ± 0.01	3.45 ± 0.05	3.45 ± 0.04	0.89	3.45 ± 0.01	3.43 ± 0.05	3.43 ± 0.04	0.66
HDL cholesterol	1.64 ± 0.01	1.63 ± 0.02	1.59 ± 0.02	0.02	1.64 ± 0.01	1.65 ± 0.02	1.63 ± 0.02	0.78
Triglycerides	1.36 ± 0.01	1.39 ± 0.05	1.41 ± 0.04	0.06 §	1.36 ± 0.01	1.37 ± 0.04	1.35 ± 0.04	0.79 §
Glycaemic status								
Glucose (mmol/L) [c]	5.88 ± 0.02	5.92 ± 0.05	5.98 ± 0.05	0.05	5.88 ± 0.02	5.91 ± 0.05	5.92 ± 0.05	0.64
Insulin (μIU/mL) [c]	8.3 ± 0.1	8.8 ± 0.4	8.8 ± 0.3	0.01 §	8.3 ± 0.1	8.5 ± 0.3	8.3 ± 0.3	0.60 §
HOMA-IR [c]	2.29 ± 0.04	2.38 ± 0.13	2.55 ± 0.12	0.04	2.3 ± 0.04	2.3 ± 0.11	2.37 ± 0.11	0.84

Diabetes	1 (ref.)	1.43 (1.06; 1.93)	1.44 (1.08; 1.91)	0.01	1 (ref.)	1.29 (0.94; 1.77)	1.22 (0.90; 1.65)	0.20
High HOMA-IR	1 (ref.)	1.21 (0.96; 1.53)	1.17 (0.94; 1.47)	0.16	1 (ref.)	1.03 (0.78; 1.35)	0.89 (0.68; 1.15)	0.36
Metabolic syndrome	1 (ref.)	1.31 (1.04; 1.64)	1.26 (1.01; 1.56)	0.04	1 (ref.)	1.15 (0.87; 1.51)	0.94 (0.72; 1.21)	0.62

For quantitative variables, multivariable analyses were performed by ANOVA and the results are expressed as adjusted mean ± standard error. For categorical variables (excluding smoking), multivariable analyses were performed by logistic regression and results are expressed as odds ratio (95% confidence interval). For smoking, multivariable analyses were performed using multivariate (polytomous) logistic regression and the results are expressed as relative risk ratio (95% confidence interval). § P-value calculated on log-transformed values. [a], adjusting for antihypertensive medication; [b], adjusting for hypolipidemic medication; [c], adjusting for antidiabetes medication.

CVRF, cardiovascular risk factors; **BMI**, body mass index; **SBP**, systolic blood pressure; **DBP**, diastolic blood pressure; **LDL**, low-density lipoprotein; **HDL**, high-density lipoprotein; **HOMA-IR**, homeostatic model assessment of insulin resistance; **high HOMA-IR** is defined as a HOMA-IR ≥2.6; **abdominal obesity** and **metabolic syndrome** are defined by the NCEP ATP-III criteria; **hypertension** is defined as SBP>140 or DBP>90 mm Hg or antihypertensive medication; **diabetes** is defined as fasting plasma glucose >7.0 mmol/L or anti-diabetes medication; **NA**, not assessable.
