# Supplementary material for: Did Dumbo suffer a heart attack? independent association between earlobe crease and cardiovascular disease
Source: BMC Cardiovasc Disord. 2016 Jan 20;16:17. doi: 10.1186/s12872-016-0193-7 (PMC4721195; doi:10.1186/s12872-016-0193-7)
Supplement: Additional file 5: Table S5. — Multivariable analysis of the association between earlobe crease and selected cardiovascular risk factors, CoLaus study, Lausanne, 2009–2012, adjusting for age, gender and waist circumference. (PDF 59 kb) [file 12872_2016_193_MOESM5_ESM.pdf]

Supplementary table 5: Multivariable analysis of the association between earlobe crease and selected cardiovascular risk factors,, CoLaus study, Lausanne, 2009-2012, adjusting for age, gender and waist circumference.

Earlobe crease	Absence (n=3829)	Presence (n=806)	P-value	Absent (n=3829)	Unilateral (n=373)	Bilateral (n=429)	P-value for trend
BMI (kg/m^2)	26.1 ± 0	26.4 ± 0.1	0.003	26.1 ± 0.1	26.4 ± 0.1	26.4 ± 0.1	0.01
Obesity	1 (ref.)	1.12 (0.82; 1.52)	0.48	1 (ref.)	1.40 (0.93; 2.12)	0.91 (0.61; 1.36)	0.64
Smoking status							
Never	-	-	-	-	-	-	-
Former	1 (ref.)	1.14 (0.95 - 1.36)	0.16	1 (ref.)	0.95 (0.75; 1.21)	1.34 (1.06; 1.70)	NA
Current	1 (ref.)	1.12 (0.90 - 1.40)	0.30	1 (ref.)	0.91 (0.67; 1.23)	1.37 (1.03; 1.82)	NA
Blood pressure status							
SBP (mm Hg) [a]	126 ± 1	126 ± 1	0.77	126 ± 1	126 ± 1	126 ± 1	0.43
DBP (mm Hg) [a]	78 ± 1	78 ± 1	0.36	78 ± 1	79 ± 1	78 ± 1	0.97
Hypertension	1 (ref.)	1.31 (1.09; 1.56)	0.004	1 (ref.)	1.34 (1.05; 1.72)	1.28 (1.01; 1.62)	0.04
Lipids (mmol/L) [b]							
Total cholesterol	5.70 ± 0.02	5.68 ± 0.04	0.53	5.70 ± 0.02	5.69 ± 0.05	5.66 ± 0.05	0.47
LDL cholesterol	3.45 ± 0.01	3.44 ± 0.03	0.66	3.45 ± 0.01	3.44 ± 0.05	3.43 ± 0.04	0.69
HDL cholesterol	1.64 ± 0.01	1.63 ± 0.01	0.69	1.64 ± 0.01	1.64 ± 0.02	1.62 ± 0.02	0.38
Triglycerides	1.36 ± 0.01	1.37 ± 0.03	0.47 §	1.36 ± 0.01	1.38 ± 0.04	1.36 ± 0.04	0.60 §
Glycaemic status							
Glucose (mmol/L) [c]	5.87 ± 0.02	5.92 ± 0.04	0.23	5.87 ± 0.02	5.92 ± 0.05	5.92 ± 0.05	0.31
Insulin (μIU/mL) [c]	8.3 ± 0.1	8.4 ± 0.2	0.43 §	8.3 ± 0.1	8.6 ± 0.3	8.3 ± 0.3	0.43 §

HOMA-IR [c]	2.3 ± 0.1	2.3 ± 0.1	0.47	2.3 ± 0.1	2.3 ± 0.1	2.4 ± 0.1	0.41
Diabetes	1 (ref.)	1.23 (0.97; 1.57)	0.08	1 (ref.)	1.28 (0.93; 1.76)	1.19 (0.88; 1.61)	0.25
High HOMA-IR	1 (ref.)	0.97 (0.79; 1.18)	0.75	1 (ref.)	1.04 (0.79; 1.36)	0.91 (0.70; 1.18)	0.46

For quantitative variables, multivariable analyses were performed by ANOVA and the results are expressed as adjusted mean ± standard error. For categorical variables (excluding smoking), multivariable analyses were performed by logistic regression and results are expressed as odds ratio (95% confidence interval). For smoking, multivariable analyses were performed using multivariate (polytomous) logistic regression and the results are expressed as relative risk ratio (95% confidence interval). § P-value calculated on log-transformed values. [a], adjusting for antihypertensive medication; [b], adjusting for hypolipidemic medication; [c], adjusting for antidiabetes medication.

BMI, body mass index; **SBP**, systolic blood pressure; **DBP**, diastolic blood pressure; **LDL**, low-density lipoprotein; **HDL**, high-density lipoprotein; **HOMA-IR**, homeostatic model assessment of insulin resistance; **high HOMA-IR** is defined as a HOMA-IR≥2.6; **hypertension** is defined as SBP>140 or DBP>90 mm Hg or antihypertensive medication; **diabetes** is defined as fasting plasma glucose >7.0 mmol/L or antidiabetes medication; **NA**, not assessable.
