# Supplementary material for: Did Dumbo suffer a heart attack? independent association between earlobe crease and cardiovascular disease
Source: BMC Cardiovasc Disord. 2016 Jan 20;16:17. doi: 10.1186/s12872-016-0193-7 (PMC4721195; doi:10.1186/s12872-016-0193-7)
Supplement: Additional file 6: Table S6. — Multivariable analysis of the association between earlobe crease and personal history of cardiovascular disease or cardiovascular risk, CoLaus study, Lausanne, 2009–2012, adjusting for age, gender and waist circumference. (PDF 51 kb) [file 12872_2016_193_MOESM6_ESM.pdf]

Supplementary table 6: Multivariable analysis of the association between earlobe crease and personal history of cardiovascular disease or cardiovascular risk, CoLaus study, Lausanne, 2009-2012, adjusting for age, gender and waist circumference.

Earlobe crease	Absence (n=3829)	Presence (n=806)	P-value	Absent (n=3829)	Unilateral (n=373)	Bilateral (n=429)	P-value for trend
History of							
Any cardiovascular disease	1 (ref.)	1.47 (1.14; 1.88)	0.003	1 (ref.)	1.44 (1.03; 2.02)	1.49 (1.09; 2.03)	0.01
Coronary artery disease	1 (ref.)	1.76 (1.23; 2.51)	0.002	1 (ref.)	1.91 (1.21; 3.01)	1.63 (1.05; 2.55)	0.03
Angina pectoris	1 (ref.)	1.16 (0.73; 1.86)	0.53	1 (ref.)	1.34 (0.73; 2.47)	1.03 (0.57; 1.87)	0.93
Myocardial infarction	1 (ref.)	1.67 (1.06; 2.62)	0.03	1 (ref.)	1.86 (1.05; 3.28)	1.50 (0.85; 2.67)	0.16
Stroke	1 (ref.)	1.28 (0.79; 2.06)	0.32	1 (ref.)	1.54 (0.84; 2.83)	1.07 (0.57; 2.01)	0.83
CABG	1 (ref.)	1.73 (0.97; 3.08)	0.06	1 (ref.)	1.79 (0.85; 3.75)	1.69 (0.84; 3.41)	0.14
High CVD risk							
SCORE recalibrated	1 (ref.)	1.08 (0.85; 1.36)	0.53	1 (ref.)	0.93 (0.67; 1.29)	1.21 (0.91; 1.63)	0.19
Framingham 1998	1 (ref.)	0.98 (0.75; 1.27)	0.85	1 (ref.)	1.02 (0.72; 1.43)	0.94 (0.67; 1.32)	0.70
Framingham 1998 r.	1 (ref.)	1.02 (0.80; 1.32)	0.86	1 (ref.)	1.07 (0.77; 1.48)	0.98 (0.70; 1.36)	0.90

Results are expressed as adjusted odds ratio (95% confidence interval) for presence relative to the absence of earlobe crease. Statistical analysis by logistic regression**. CVD**, cardiovascular disease; **CABG**, Coronary artery bypass graft; **high CVD risk** is defined as a 10-year risk ≥5% for SCORE and as a 10-year risk ≥20% for Framingham. r. recalibrated.
