# Supplementary material for: Did Dumbo suffer a heart attack? independent association between earlobe crease and cardiovascular disease
Source: BMC Cardiovasc Disord. 2016 Jan 20;16:17. doi: 10.1186/s12872-016-0193-7 (PMC4721195; doi:10.1186/s12872-016-0193-7)
Supplement: Additional file 7: Table S7. — Multivariable analysis of the association between earlobe crease and personal history of cardiovascular disease, CoLaus study, Lausanne, 2009–2012, adjusted for age, gender, waist circumference, total cholesterol, hypertension, diabetes and smoking. (PDF 46 kb) [file 12872_2016_193_MOESM7_ESM.pdf]

Supplementary table 7: Multivariable analysis of the association between earlobe crease and personal history of cardiovascular disease, CoLaus study, Lausanne, 2009-2012, adjusted for age, gender, waist circumference, total cholesterol, hypertension, diabetes and smoking.

Earlobe crease	Absence (n=3829)	Presence (n=806)	P-value	Absent (n=3829)	Unilateral (n=373)	Bilateral (n=429)	P-value for trend
Any cardiovascular disease	1 (ref.)	1.36 (1.05; 1.77)	0.02	1 (ref.)	1.39 (0.97; 1.97)	1.34 (0.97; 1.86)	0.08
Coronary artery disease	1 (ref.)	1.63 (1.12; 2.36)	0.01	1 (ref.)	1.90 (1.18; 3.06)	1.42 (0.89; 2.27)	0.14
Angina pectoris	1 (ref.)	1.05 (0.65; 1.69)	0.84	1 (ref.)	1.25 (0.67; 2.33)	0.91 (0.50; 1.67)	0.76
Myocardial infarction	1 (ref.)	1.55 (0.96; 2.48)	0.07	1 (ref.)	1.87 (1.04; 3.37)	1.30 (0.71; 2.37)	0.40
Stroke	1 (ref.)	1.21 (0.74; 1.96)	0.44	1 (ref.)	1.49 (0.81; 2.74)	1.00 (0.53; 1.88)	1.00
Coronary artery bypass graft	1 (ref.)	1.63 (0.90; 2.95)	0.11	1 (ref.)	1.82 (0.85; 3.90)	1.49 (0.72; 3.09)	0.28

Results are expressed as adjusted odds ratio (95% confidence interval) for presence relative to the absence of earlobe crease. Statistical analysis by logistic regression**.**
