# Supplementary material for: Did Dumbo suffer a heart attack? independent association between earlobe crease and cardiovascular disease
Source: BMC Cardiovasc Disord. 2016 Jan 20;16:17. doi: 10.1186/s12872-016-0193-7 (PMC4721195; doi:10.1186/s12872-016-0193-7)
Supplement: Additional file 8: Table S8. — Bivariate association between earlobe crease (unilateral or bilateral) and history of cardiovascular disease, CoLaus study, Lausanne, 2009–2012. (PDF 39 kb) [file 12872_2016_193_MOESM8_ESM.pdf]

Supplementary table 8: Bivariate association between earlobe crease (unilateral or bilateral) and history of cardiovascular disease, CoLaus study, Lausanne, 2009-2012.

**Earlobe crease**	**Absent (n=3829)**	**Unilateral (n=373)**	**Bilateral (n=429)**	**P-value**
Any cardiovascular disease	244 (6.4)	52 (13.9)	70 (16.2)	<0.001
Coronary artery disease	92 (2.4)	28 (7.5)	32 (7.4)	<0.001
Angina pectoris	61 (1.6)	14 (3.8)	16 (3.7)	<0.001
Myocardial infarction	60 (1.6)	17 (4.6)	17 (3.9)	<0.001
Stroke	61 (1.6)	14 (3.8)	14 (3.3)	<0.001
Coronary artery bypass graft	33 (0.9)	10 (2.7)	12 (2.8)	<0.001

Results are expressed as number of participants (column %). Statistical analysis by chi-square.
