# Supplementary material for: Did Dumbo suffer a heart attack? independent association between earlobe crease and cardiovascular disease
Source: BMC Cardiovasc Disord. 2016 Jan 20;16:17. doi: 10.1186/s12872-016-0193-7 (PMC4721195; doi:10.1186/s12872-016-0193-7)
Supplement: Additional file 9: Table S9. — Multivariable association between earlobe crease (unilateral or bilateral) and history of cardiovascular disease, CoLaus study, Lausanne, 2009–2012. (PDF 48 kb) [file 12872_2016_193_MOESM9_ESM.pdf]

Supplementary table 9: Multivariable association between earlobe crease (unilateral or bilateral) and history of cardiovascular disease, CoLaus study, Lausanne, 2009-2012.

Adjusted for	Age and gender				Age, gender and body mass index			
Earlobe crease	Absent (n=3829)	Unilateral (n=373)	Bilateral (n=429)	P-value for trend	Absent (n=3829)	Unilateral (n=373)	Bilateral (n=429)	P-value for trend
Any CVD	1 (ref.)	1.48 (1.06; 2.07)	1.61 (1.18; 2.18)	<0.01	1 (ref.)	1.42 (1.01; 2.00)	1.50 (1.10; 2.06)	0.01
Coronary artery disease	1 (ref.)	1.95 (1.24; 3.07)	1.76 (1.14; 2.73)	0.01	1 (ref.)	1.93 (1.22; 3.04)	1.70 (1.09; 2.65)	0.02
Angina pectoris	1 (ref.)	1.41 (0.77; 2.59)	1.18 (0.66; 2.10)	0.58	1 (ref.)	1.35 (0.73; 2.49)	1.06 (0.59; 1.93)	0.84
Myocardial infarction	1 (ref.)	1.86 (1.06; 3.27)	1.53 (0.87; 2.71)	0.15	1 (ref.)	1.69 (0.95; 3.03)	1.51 (0.85; 2.68)	0.16
Stroke	1 (ref.)	1.56 (0.85; 2.85)	1.17 (0.64; 2.15)	0.61	1 (ref.)	1.60 (0.87; 2.95)	1.06 (0.55; 2.02)	0.87
CABG	1 (ref.)	1.74 (0.83; 3.62)	1.67 (0.83; 3.35)	0.15	1 (ref.)	1.58 (0.73; 3.41)	1.74 (0.86; 3.52)	0.12

Results are expressed as age and gender-adjusted odds ratio (95% confidence interval). Statistical analysis by logistic regression.

CVD, cardiovascular disease; **CABG**, coronary artery bypass graft.
