# Supplementary material for: Did Dumbo suffer a heart attack? independent association between earlobe crease and cardiovascular disease
Source: BMC Cardiovasc Disord. 2016 Jan 20;16:17. doi: 10.1186/s12872-016-0193-7 (PMC4721195; doi:10.1186/s12872-016-0193-7)
Supplement: Additional file 10: Table S10. — Bivariate analysis of the association between earlobe crease and physical activity, adipokines or liver markers, CoLaus study, Lausanne, 2009–2012. (PDF 43 kb) [file 12872_2016_193_MOESM10_ESM.pdf]

Supplementary table 10: Bivariate analysis of the association between earlobe crease and physical activity, adipokines or liver markers, CoLaus study, Lausanne, 2009-2012.

Earlobe crease	Absence (n=3829)	Presence (n=806)	P-value
TEE (kcal/day)	2686 ± 641	2679 ±625	0.78
Sedentary (%)	1833 (57.0)	378 (58.9)	0.38
Adipokines (ng/mL) §			
Leptin	4813 ± 6099	5530 ± 7386	<0.05
Adiponectin	4962 ± 4291	4860 ± 4195	0.37
Uric acid (mmol/L)	308 ± 79	335 ± 87	<0.001
Creatinine (μmol/L)	79.2 ± 18.6	83.7 ± 18.0	<0.001
Liver enzymes (IU/L) §			
ASAT	28.9 ± 11.8	29.7 ± 10.9	<0.01
ALAT	27.5 ± 17.6	28.5 ± 16.1	<0.01
γ-GT	36.0 ± 46.7	43.7 ± 55.2	<0.001
Alkaline phosphatase	62.4 ± 19.9	64.5 ± 20.5	<0.01

Results are expressed as number of participants (%) or as mean ± standard deviation.

Statistical analysis by Student's t-test. § P-value calculated on log-transformed values.

TEE, total energy expenditure; **ASAT**, aspartate aminotransferase; **ALAT**, alanine aminotransferase; **γ-GT**, γ-glutamyl transpeptidase; **Sedentary** is defined as expending less than 10% of the daily energy in moderate- and high-intensity activities (at least 4 times the basal metabolic rate).
