# Supplementary material for: Did Dumbo suffer a heart attack? independent association between earlobe crease and cardiovascular disease
Source: BMC Cardiovasc Disord. 2016 Jan 20;16:17. doi: 10.1186/s12872-016-0193-7 (PMC4721195; doi:10.1186/s12872-016-0193-7)
Supplement: Additional file 11: Table S11. — Multivariable analysis of the association between ELC and physical activity, adipokines or liver markers, CoLaus study, Lausanne, 2009–2012. (PDF 54 kb) [file 12872_2016_193_MOESM11_ESM.pdf]

Supplementary table 11: Multivariable analysis of the association between ELC and physical activity, adipokines or liver markers, CoLaus study, Lausanne, 2009-2012.

	**Adjusted for age and gender**			**Adjusted for age, gender and body mass index**		
**Earlobe crease**	**Absence (n=3829)**	**Presence (n=806)**	**P-value**	**Absence (n=3829)**	**Presence (n=806)**	**P-value**
TEE (kcal/day)	2683±9	2698±20	0.47	2688±8	2671±19	0.39
Sedentary	1 (ref.)	0.94 (0.78; 1.13)	0.50	1 (ref.)	0.89 (0.74; 1.07)	0.20
Adipokines (ng/mL)						
Leptin	4791 ± 99	5637 ± 225	<0.001 §	4916 ± 76	5016 ± 173	0.70 §
Adiponectin	4984 ± 68	4756 ± 31	0.15 §	4953 ± 67	4843 ± 153	0.48 §
Uric acid (mmol/L)	311 ± 1.1	318 ± 2.5	0.01	312 ± 1.1	314 ± 2.4	0.46
Creatinine (µmol/L)	79.9 ±0.3	80.4 ± 0.6	0.48	79.9 ± 0.3	80.4 ± 0.6	0.53
Liver enzymes (IU/L)						
ASAT	29.0 ± 0.2	28.8 ± 0.4	0.71 §	29.1 ± 0.2	28.6 ± 0.4	0.39 §
ALAT	27.6 ± 0.3	28.0 ± 0.6	0.17 §	27.8 ± 0.3	27.4 ± 0.6	0.89 §
γ-GT	36.9 ± 0.8	39.6 ± 1.7	0.09 §	37.1 ± 0.8	38.9 ± 1.7	0.39 §
Alkaline phosphatase	62.9 ± 0.3	62.1 ± 0.7	0.44 §	63.0 ± 0.3	61.6 ± 0.7	0.12 §

Results are expressed as adjusted mean ± standard error for quantitative variables and as odds ratio (95% confidence interval) for categorical variables. Statistical analysis by ANOVA for quantitative variables and by logistic regression for categorical variables. § P-value calculated on log-transformed values.

TEE, total energy expenditure; **ASAT**, aspartate aminotransferase; **ALAT**, alanine aminotransferase; **γ-GT**, γ-glutamyl transpeptidase; **Sedentary** is defined as expending less than 10% of the daily energy in moderate- and high-intensity activities (at least 4 times the basal metabolic rate).
