# Supplementary material for: Did Dumbo suffer a heart attack? independent association between earlobe crease and cardiovascular disease
Source: BMC Cardiovasc Disord. 2016 Jan 20;16:17. doi: 10.1186/s12872-016-0193-7 (PMC4721195; doi:10.1186/s12872-016-0193-7)
Supplement: Additional file 12: Table S12. — Multivariable analysis of the association between earlobe crease and physical activity, adipokines or liver markers, CoLaus study, Lausanne, 2009–2012, adjusted for age, gender and waist circumference. (PDF 55 kb) [file 12872_2016_193_MOESM12_ESM.pdf]

Supplementary table 12: Multivariable analysis of the association between earlobe crease and physical activity, adipokines or liver markers, CoLaus study, Lausanne, 2009-2012, adjusted for age, gender and waist circumference.

**Earlobe crease**	**Absence (n=3829)**	**Presence (n=806)**	**P-value**	**Absent (n=3829)**	**Unilateral (n=373)**	**Bilateral (n=429)**	**P-value for trend**
TEE (kcal/day)	2685 ± 8	2679 ± 19	0.76	2686 ± 8	2707 ± 27	2653 ± 26	0.24
Sedentary	1 (ref.)	0.89 (0.74; 1.07)	0.23	1 (ref.)	0.74 (0.58; 0.95)	1.07 (0.83; 1.37)	0.60
Adipokines (ng/mL)							
Leptin	4870 ± 80	5133 ± 181	0.24 §	4870 ± 80	5144 ± 259	5124 ± 247	0.67 §
Adiponectin	4965 ± 67	4850 ± 153	0.47 §	4965 ± 67	4770 ± 218	4924 ± 208	0.64 §
Uric acid (mmol/L)	312 ± 1	315 ± 2	0.27	312 ± 1	319 ± 3	311 ± 3	0.72
Creatinine (μmol/L)	80 ± 1	80 ± 1	0.53	80 ± 1	80 ± 1	80 ± 1	0.53
Liver enzymes (IU/L)							
ASAT	29.1 ± 0.2	28.6 ± 0.4	0.35 §	29.1 ± 0.2	28.8 ± 0.6	28.5 ± 0.6	0.22 §
ALAT	27.7 ± 0.3	27.4 ± 0.6	0.88 §	27.7 ± 0.3	27.6 ± 0.8	27.3 ± 0.8	0.49 §
γ-GT	37.0 ± 0.8	38.6 ± 1.7	0.49 §	37.0 ± 0.8	40.5 ± 2.4	37.0 ± 2.3	0.64 §
Alkaline phosphatase	63.0 ± 0.3	61.6 ± 0.7	0.10 §	63.0 ± 0.3	61.9 ± 1.0	61.3 ± 0.9	0.23 §

Results are expressed as adjusted mean ± standard error for quantitative variables and as odds ratio (95% confidence interval) for categorical variables. Statistical analysis by ANOVA for quantitative variables and by logistic regression for categorical variables. § P-value calculated on log-transformed values.

TEE, total energy expenditure; **ASAT**, aspartate aminotransferase; **ALAT**, alanine aminotransferase; **γ-GT**, γ-glutamyl transpeptidase; **Sedentary** is defined as expending less than 10% of the daily energy in moderate- and high-intensity activities (at least 4 times the basal metabolic rate).
