# Supplementary material for: Did Dumbo suffer a heart attack? independent association between earlobe crease and cardiovascular disease
Source: BMC Cardiovasc Disord. 2016 Jan 20;16:17. doi: 10.1186/s12872-016-0193-7 (PMC4721195; doi:10.1186/s12872-016-0193-7)
Supplement: Additional file 13: Table S13. — Bivariate association between earlobe crease and quartiles of inflammatory markers, CoLaus study, Lausanne, 2009–2012. (PDF 41 kb) [file 12872_2016_193_MOESM13_ESM.pdf]

Supplementary table 13: Bivariate association between earlobe crease and quartiles of inflammatory markers, CoLaus study, Lausanne, 2009-2012.

Earlobe crease	Absence (n=3829)	Presence (n=806)	P-value
High sensitivity C-reactive protein			
First	1177 (30.9)	175 (21.8)	
Second	827 (21.7)	185 (23.0)	<0.001
Third	925 (24.2)	213 (26.5)	
Fourth	884 (23.2)	231 (28.7)	
Interleukin-1β			
First	930 (26.1)	190 (26.1)	
Second	824 (23.1)	170 (23.4)	0.58
Third	910 (25.6)	170 (23.4)	
Fourth	899 (25.2)	197 (27.1)	
Interleukin-6			
First	888 (24.9)	193 (26.5)	
Second	886 (24.8)	182 (25.0)	0.82
Third	901 (25.3)	178 (24.4)	
Fourth	890 (25.0)	176 (24.1)	
Tumour necrosis factor-α			
First	927 (26.0)	155 (21.3)	
Second	890 (24.9)	179 (23.3)	<0.05
Third	869 (24.4)	201 (27.6)	
Fourth	881 (24.7)	203 (27.8)	

Results are expressed as number of participants (%). Statistical analysis by chi-square.
