# Supplementary material for: Did Dumbo suffer a heart attack? independent association between earlobe crease and cardiovascular disease
Source: BMC Cardiovasc Disord. 2016 Jan 20;16:17. doi: 10.1186/s12872-016-0193-7 (PMC4721195; doi:10.1186/s12872-016-0193-7)
Supplement: Additional file 14: Table S14. — Multivariable association between earlobe crease and being in the highest quartile of inflammatory markers, CoLaus study, Lausanne, 2009–2012. (PDF 51 kb) [file 12872_2016_193_MOESM14_ESM.pdf]

Supplementary table 14: Multivariable association between earlobe crease and being in the highest quartile of inflammatory markers, CoLaus study, Lausanne, 2009-2012.

	Adjusted for age and gender			Adjusted for age, gender and body mass index		
**Earlobe crease**	**Absence (n=3829)**	**Presence (n=806)**	**P-value**	**Absence (n=3829)**	**Presence (n=806)**	**P-value**
Quartiles						
Hs-CRP	1 (ref.)	1.19 (1.00; 1.42)	0.056	1 (ref.)	1.04 (0.86; 1.26)	0.66
Interleukin-1β	1 (ref.)	1.22 (1.01; 1.48)	0.04	1 (ref.)	1.23 (1.02; 1.49)	0.03
Interleukin-6	1 (ref.)	0.98 (0.81; 1.19)	0.84	1 (ref.)	0.98 (0.81; 1.19)	0.87
TNF- α	1 (ref.)	1.08 (0.90; 1.30)	0.81	1 (ref.)	1.04 (0.86; 1.25)	0.70
Log-transformed values §						
Hs-CRP (mg/dL)	1.37 ± 1.02	1.52 ± 1.04	0.02	1.39 ± 1.02	1.41 ± 1.04	0.73
Interleukin-1β (pg/mL)	1.49 ± 1.03	1.65 ± 1.07	0.15	1.49 ± 1.03	1.65 ± 1.07	0.14
Interleukin-6 (pg/mL)	3.53 ± 1.03	3.24 ± 1.06	0.22	3.55 ± 1.03	3.24 ± 1.07	0.19
TNF-α (pg/mL)	4.70 ± 1.02	4.83 ± 1.04	0.53	4.74 ± 1.02	4.77 ± 1.04	0.87

Results are expressed as adjusted mean ± standard error for quantitative variables and as odds ratio (95% confidence interval) for categorical variables. Statistical analysis by ANOVA for quantitative variables and by logistic regression for categorical variables. § excluding participants with undetectable values. **Hs-CRP**, high sensitivity C-reactive protein; **TNF-α**, tumour necrosis factor alpha.
