# Supplementary material for: Did Dumbo suffer a heart attack? independent association between earlobe crease and cardiovascular disease
Source: BMC Cardiovasc Disord. 2016 Jan 20;16:17. doi: 10.1186/s12872-016-0193-7 (PMC4721195; doi:10.1186/s12872-016-0193-7)
Supplement: Additional file 15: Table S15. — Multivariable association between earlobe crease and being in the highest quartile of inflammatory markers, CoLaus study, Lausanne, 2009–2012, adjusted for age, gender and waist circumference. (PDF 57 kb) [file 12872_2016_193_MOESM15_ESM.pdf]

Supplementary table 15: Multivariable association between earlobe crease and being in the highest quartile of inflammatory markers, CoLaus study, Lausanne, 2009-2012, adjusted for age, gender and waist circumference.

Earlobe crease	Absence (n=3829)	Presence (n=806)	P-value	Absent (n=3829)	Unilateral (n=373)	Bilateral (n=429)	P-value for trend
Quartiles							
Hs-CRP	1 (ref.)	1.06 (0.87; 1.27)	0.58	1 (ref.)	1.17 (0.90; 1.51)	0.97 (0.76; 1.23)	0.78
Interleukin-1β	1 (ref.)	1.22 (1.01; 1.48)	0.04	1 (ref.)	1.16 (0.90; 1.50)	1.28 (1.00; 1.63)	0.05
Interleukin-6	1 (ref.)	0.98 (0.81; 1.19)	0.82	1 (ref.)	0.96 (0.73; 1.25)	1.00 (0.77; 1.28)	0.98
TNF-α	1 (ref.)	1.05 (0.87; 1.27)	0.59	1 (ref.)	1.11 (0.87; 1.43)	1.00 (0.78; 1.28)	1.00
Log-transformed values §							
Hs-CRP (mg/dL)	0.33 ± 0.02	0.35 ± 0.03	0.50	0.33 ± 0.02	0.40 ± 0.05	0.31 ± 0.05	0.69
Interleukin-1β (pg/mL)	0.40 ± 0.03	0.51 ± 0.07	0.15	0.40 ± 0.03	0.53 ± 0.09	0.49 ± 0.09	0.36
Interleukin-6 (pg/mL)	1.26 ± 0.03	1.17 ± 0.06	0.17	1.26 ± 0.03	1.14 ± 0.09	1.19 ± 0.09	0.44
TNF-α (pg/mL)	1.55 ± 0.02	1.56 ± 0.04	0.83	1.55 ± 0.02	1.57 ± 0.06	1.56 ± 0.05	0.95

Results are expressed as adjusted mean ± standard error for quantitative variables and as odds ratio (95% confidence interval) for categorical variables. Statistical analysis by ANOVA for quantitative variables and by logistic regression for categorical variables. § excluding participants with undetectable values. **Hs-CRP**, high sensitivity C-reactive protein; **TNF-α**, tumour necrosis factor alpha.
